# Pathogenicity of the root lesion nematode *Pratylenchus neglectus* depends on pre-culture conditions

**DOI:** 10.1038/s41598-023-46551-9

**Published:** 2023-11-10

**Authors:** Ehsan Fatemi, Christian Jung

**Affiliations:** Plant Breeding Institute, Christian-Albrechts University, Kiel, Germany

**Keywords:** Ecology, Microbiology, Molecular biology, Plant sciences, Zoology, Ecology, Environmental sciences

## Abstract

The ability of a plant parasitic nematode to infect and reproduce within a host plant depends on its genotype and the environmental conditions before and during infection. We studied the culturing conditions of the root lesion nematode *Pratylenchus neglectus* to produce inoculum for plant infection tests. Nematodes were either cultivated on carrot calli for different periods or directly isolated from the roots of the host plants. After infection of wheat and barley plants in the greenhouse, nematodes were quantified by RT-qPCR and by visual counting of the nematodes. We observed drastically reduced infection rates after long-term (> 96 weeks) cultivation on carrot callus. In contrast, fresh isolates from cereal roots displayed much higher pathogenicity. We recommend using root lesion nematodes cultivated on carrot calli no longer than 48 weeks to guarantee uniform infection rates.

## Introduction

Root lesion nematodes (RLN) of the genus *Pratylenchus* are the world's third most significant plant parasitic nematodes (PPN) after root-knot and cyst nematodes^1^. Four species causing high yield losses in a wide range of crops (*P. crenatus*, *P. neglectus*, *P. penetrans*, and *P. thornei*) are abundant in different climate zones around the globe. In temperate regions, *P. neglectus* and *P. thornei* are major threats to cereal production. They often occur as a mixed population^2–4^, primarily feeding on plant roots. Upon infection, necrotic lesions are formed that can reduce the plant's ability to absorb nutrients, which, in combination with secondary infections by other pathogens, can result in stunted growth, wilting, and reduced yield^1,2,5^. The severity of *Pratylenchus* infestations depends on the nematode species' composition, crop rotations, environmental conditions, and the nematode's pathogenicity^2^.

A standard inoculum is used for testing large plant populations during plant breeding. Appropriate methods for cultivating *Pratylenchus* spp. were established by Mountain^6^ for peach and tobacco. Since then, the monoxenic culture on carrot calli has been commonly used^2,6^. The published protocols differ only by flame or chemical sterilization of carrot discs or the chemicals used for surface sterilization of nematodes (HgCl_2_ or streptomycin sulfate) and their concentration^7–10^.

The cultivation on carrot calli aims to multiply nematodes by maintaining their genotype and pathogenicity. The pathogenicity is composed of fitness and virulence^11^, defined as the nematode's capacity to initiate a host-parasite interaction and its ability to inflict damage^2^. Fitness refers to the reproduction rate in a given environment. Virulence, in this respect, has been defined as the severity of infection, whereas aggressiveness refers to the ability of a nematode to compete with other nematodes or parasites for resources within the host^11–13^. The pathogenicity of *Pratylenchus* species is influenced by their virulence and fitness, by the host's vigorousness, e.g., due to nutrient supply, and by edaphic factors such as soil texture and temperature, and the interaction with the microbiome^2,11,12,14–18^. Although no *Pratylenchus* spp. races have been reported so far, host compatibility and pathogenicity in vegetable and fruit tree crops vary between isolates from different geographical regions^2,19,20^.

The interaction between nematodes and their microbiomes has been well-studied in the past years^21–27^. Dirksen, et al.^22^ discovered a species-rich bacterial community associated with *Caenorhabditis elegans* inside its pharynx (oesophagus), dominated by Proteobacteria such as Enterobacteriaceae of the genera *Pseudomonas*, *Stenotrophomonas*, *Ochrobactrum*, and *Sphingomonas*. The associated microbiome can have a direct impact on host development by supplying food and necessary metabolic compounds^28^ or by working as a stimulus for morphogenesis and/or pathogenicity^22,26,27,29^. Thus, the bacterial community can improve the nematode's fitness, especially under stress conditions. Another study with *P. neglectus* found that various bacterial species attached to the cuticle influenced the interactions with the host plant. In particular, the presence of *Rothia* sp. decreased the penetrating efficiency and pathogenicity in barley roots^26^.

This project aimed to assess the pathogenicity of *P. neglectus* after cultivation under different environments. For infection of cereal roots, we used nematodes cultivated on carrot callus and barley and wheat roots. Low infection rates suggested that long-term cultivation on carrot callus drastically reduced the pathogenicity of root lesion nematodes. This study demonstrates that infection rates can be severely flawed by inappropriate inoculum. Thus, the results are vital for breeding resistant cereal varieties.

## Results

**Figure 1:**
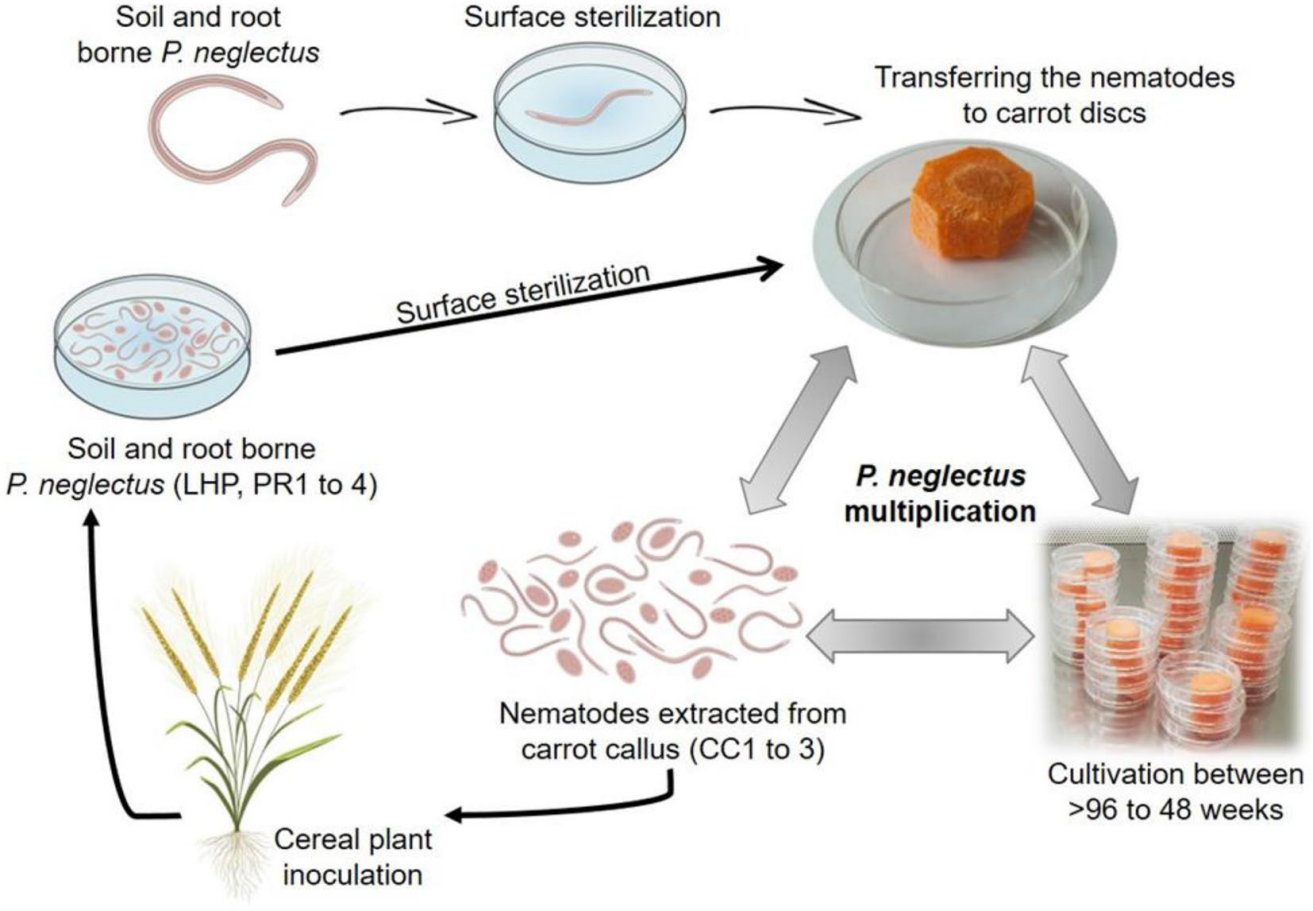
Experimental design and production of *P. neglectus* inoculum from different origins. The susceptible barley variety Valentina and the susceptible wheat variety Machete were utilized. The experiments were performed twice, with 30 biological replicates. The nematode isolates were obtained between > 96 to 48 weeks on carrot callus (isolates CC1, CC2, CC3) or from barley and wheat roots (LHP and PR1, PR2, PR3, PR4) (Table 2). Ten days after sowing, the plants were inoculated with 1000 nematodes and grown in the greenhouse under long-day conditions (16 h of light) at 23 °C during the day and 18 °C at night. The plants were harvested eight weeks after inoculation. Non-inoculated plants were used as controls.

We aimed to investigate the nematode's pathogenicity after growth under different environments. We suspected extended pre-culture might decrease pathogenicity. Therefore, *P. neglectus* was grown under monoxenic culture conditions on carrot callus and quasi-natural conditions on barley and wheat roots. The monoxenic carrot culture (CC1, CC2, and CC3) isolates were obtained after cultivation between > 96 and 48 weeks. The LHP isolate was maintained for 48 weeks on living host plants and extracted directly from barley and wheat roots infected with *P. neglectus*. The PR1 to 4 isolates were also obtained from infected barley and wheat roots with CC1 to 3 and LHP isolates (Fig. 1 and Table 2).

As a result, all morphological features aligned with the common descriptors of *P. neglectus* (data not shown). Moreover, species identity was determined by the species-specific primer combination Neg1. The PCR yielded the expected 234 bp amplicon (Supplementary Fig. 1A). The RT-qPCR assay showed the expected amplification curves and a single melting peak at 81.5 °C (Supplementary Fig. 1B, C)^30^.

**Figure 2:**
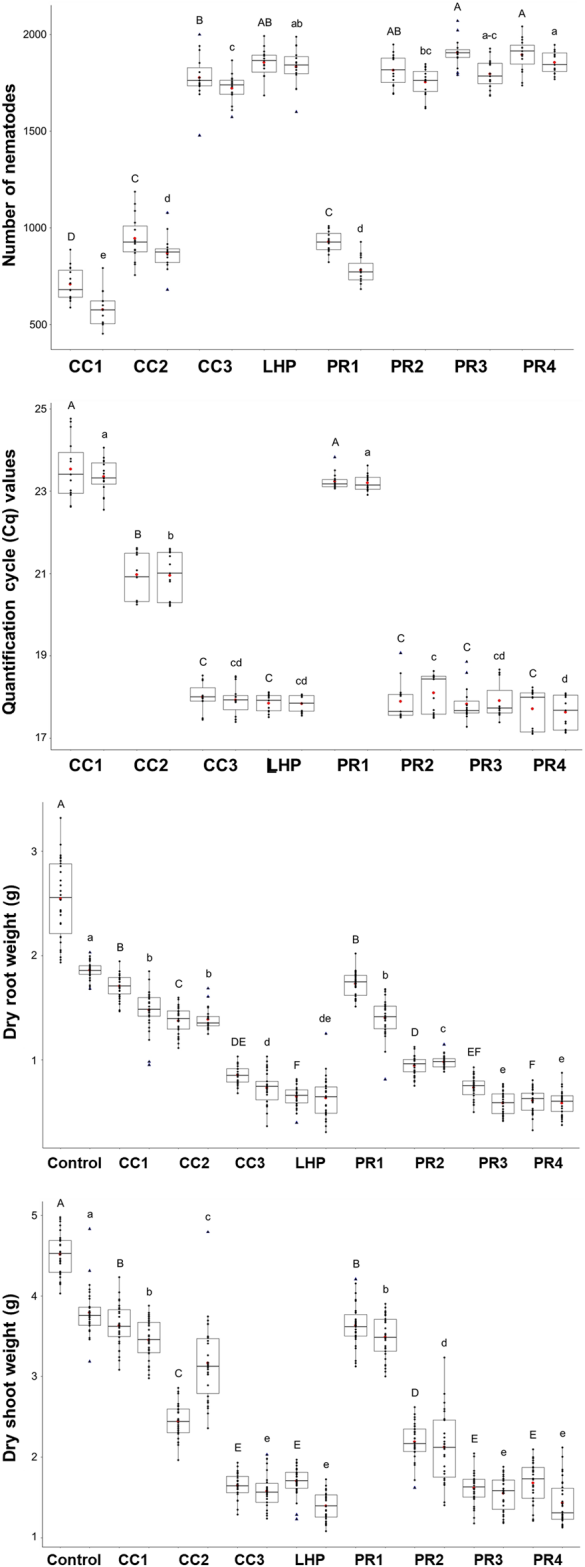

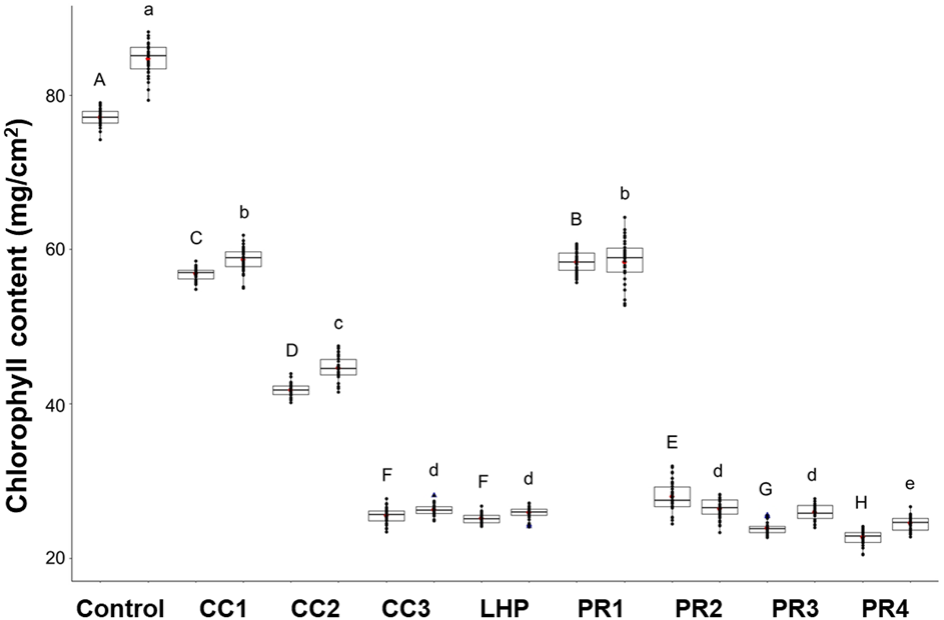
Results from greenhouse infection tests with wheat and barley to measure the effect of nematode pre-cultivation on the pathogenicity. Plants were inoculated with 1000 nematodes (Pi) from different *P. neglectus* isolates (CC1, CC2, CC3, LHP, and PR1, PR2, PR3, PR4, see Fig. 1 and Table 2) and harvested eight weeks later. For the RT-qPCR detection assay, the primer combination Neg1 was used. Black and red dots are the individual and mean values, respectively. The upper and lower quartiles are separated by the median (horizontal line). Blue triangles represent outliers. Error bars represent the standard error of the mean from biological replicates. An ANOVA test (p < 0.05) was performed, and significant differences between groups were calculated by a Tukey test (p < 0.05). Different letters above error bars represent groups based on significance (A–H: barley; a–h: wheat).

The roots were harvested eight weeks after infection. The number of nematodes per barley and wheat plant after infection with CC and LHP isolates ranged from 708.24 ± 83.46 to 1853.29 ± 72.4 (Pf/Pi from 0.71 to 1.85) and from 576.07 ± 86.23 to 1831.08 ± 96.2 (Pf/Pi from 0.58 to 1.83), respectively (Fig. 2; Table 1). After infection with PR isolates, the number of nematodes ranged from 926.38 ± 54.13 to 1894.23 ± 83.07 (Pf/Pi from 0.93 to 1.89) and from 779.62 ± 65.09 to 1853.99 ± 55.65 (Pf/Pi from 0.78 to 1.85), respectively. The plants inoculated with short-term isolates from carrot callus (CC3) and cereal roots (LHP, PR2, PR3, and PR4) displayed the highest Pf/Pi ratios. In contrast, plants infected with long-term isolates from carrot callus (CC1, CC2, and PR1) displayed the lowest Pf/Pi ratios (Fig. 2, number of nematodes; Table 1).

**Table 1:** Results from infection experiment with different *P. neglectus* isolates.

Nematode inoculum	Host species	Number of nematodes/plant by visual counting				Cq values		
		Mean ± SD	(Min–max)	CV	Pf/Pi	Mean ± SD	(Min–max)	CV
CC1	Barley	708.2 ± 83.5	(588–886)	11.78	0.71	23.54 ± 0.70	(22.62–24.76)	2.99
	Wheat	576.1 ± 86.2	(451–791)	14.97	0.58	23.36 ± 0.40	(22.55–24.06)	1.71
CC2	Barley	943.2 ± 116.4	(756–1187)	12.34	0.94	20.97 ± 0.54	(20.25–21.62)	2.56
	Wheat	867.4 ± 87.3	(679–1076)	10.06	0.87	20.95 ± 0.55	(20.21–21.60)	2.64
CC3	Barley	1776.7 ± 118.7	(1477–1999)	6.68	1.78	18.00 ± 0.33	(17.45–18.52)	1.85
	Wheat	1720.6 ± 73.4	(1572–1866)	4.26	1.72	17.93 ± 0.35	(17.40–18.51)	1.94
LHP	Barley	1853.3 ± 72.4	(1683–1991)	3.91	1.85	17.85 ± 0.21	(17.51–18.12)	1.16
	Wheat	1831.1 ± 96.2	(1599–1987)	5.25	1.83	17.83 ± 0.20	(17.55–18.06)	1.13
PR1	Barley	926.4 ± 54.1	(822–1008)	5.84	0.93	23.24 ± 0.20	(23.07–23.82)	0.85
	Wheat	779.6 ± 65.1	(682–927)	8.35	0.78	23.20 ± 0.19	(22.91–23.62)	0.82
PR2	Barley	1813.9 ± 77.2	(1692–1948)	4.26	1.81	17.90 ± 0.44	(17.50–19.07)	2.46
	Wheat	1753.1 ± 68.9	(1618–1846)	3.93	1.75	18.10 ± 0.45	(17.50–18.63)	2.50
PR3	Barley	1905.4 ± 71.1	(1790–2069)	3.73	1.91	17.83 ± 0.40	(17.28–18.86)	2.27
	Wheat	1794.2 ± 74.4	(1683–1927)	4.15	1.79	17.91 ± 0.43	(17.39–18.67)	2.40
PR4	Barley	1894.2 ± 83.1	(1735–2041)	4.39	1.89	17.71 ± 0.46	(17.10–18.24)	2.63
	Wheat	1854.1 ± 55.7	(1768–1947)	3.00	1.85	17.63 ± 0.40	(17.13–18.09)	2.26

Roots were harvested 8 weeks after inoculation. Root samples were divided into two groups for visual counting (displayed as the number of nematodes) and RT-qPCR. The quantification cycle (Cq) values were obtained after RT-qPCR with the species-specific primer combination Neg1. Isolates are described in Fig. 1 and Table 2. Nematodes were counted under a stereo microscope. The average multiplication rate was determined by dividing the final number of nematodes by the initial number of nematodes (Pf/Pi).

*CV* coefficient of variation (%).

**Table 2:** The origin of nematode isolates used in this study.

Nematode isolates	Pre-cultivation on host plant/tissue	Weeks of pre-cultivation	Host plant inoculated	Total number of inoculated host plants
CC1	Carrot callus	> 96	Barley and wheat	60
CC2	Carrot callus	96	Barley and wheat	60
CC3	Carrot callus	48	Barley and wheat	60
LHP	Barley and Wheat	48	Barley and wheat	60
PR1	CC1 on barley and wheat	8 weeks	Barley and wheat	60
PR2	CC2 on barley and wheat	8 weeks	Barley and wheat	60
PR3	CC3 on barley and wheat	8 weeks	Barley and wheat	60
PR4	LHP on barley and wheat	8 weeks	Barley and wheat	60

The susceptible barley and wheat varieties Valentina and Machete served as host plants, respectively. The experiments were performed twice, with 30 barley and wheat plants. Ten days after sowing, the plants were inoculated with 1000 *P. neglectus* nematodes (Pi) and grown in the greenhouse under long-day conditions (16 h of light) at 23 °C during the day and 18 °C at night. The plants were harvested eight weeks after inoculation. Non-inoculated barley and wheat plants served as controls (Fig. 1).

Then, we measured the abundance of nematode DNA within the infected roots using our recently established RT-qPCR detection assay. The quantification cycle (Cq) values for barley and wheat plants infected with CC and LHP ranged from 23.54 ± 0.7 to 17.85 ± 0.21 and from 23.36 ± 0.4 to 17.83 ± 0.2, respectively. The Cq values after infection with PR isolates were strikingly lower, ranging from 23.24 ± 0.2 to 17.71 ± 0.46 for barley and 23.2 ± 0.19 to 17.63 ± 0.40 for wheat (Fig. 2, Cq values; Table 1). These results correspond with previous findings that showed a negative correlation between the number of nematodes and the quantification cycle (Cq) values^30^. In conclusion, nematodes pre-cultured on carrot calli were less infective than those grown on cereal roots. We expected a correlation between infection rates and plant growth. Therefore, we measured the biomass and chlorophyll content eight weeks after inoculation (Supplementary Fig. 2). We reasoned that isolates with higher pathogenicity resulted in heavy infections, causing biomass and chlorophyll content reductions. As a result, the biomass and the chlorophyll content varied greatly in response to different isolates. The root and shoot weight of plants infected with short-term carrot callus and cereal-grown isolates (CC3, LHP, PR2, PR3, and PR4) was significantly lower, as compared to plants infected with isolates long-grown on carrot callus (CC1, CC2, and PR1), (Fig. 2). Likewise, the chlorophyll content of plants infected with short-term carrot callus and cereal-grown isolates (CC3, LHP, PR2, PR3, and PR4) was significantly lower, as compared to plants infected with isolates long grown on carrot callus (CC1, CC2, and PR1) (Fig. 2, chlorophyll content). Thus, there was a negative correlation between the number of nematodes per root and biomass/chlorophyll content.

## Discussion

The multiplication and maintenance of root lesion nematodes is challenging because they do not form durable stages like cyst nematodes. We assessed different pre-cultivation methods to produce inoculum for plant infection experiments. Long-term cultivation on carrot callus drastically reduced the nematode's pathogenicity, i.e., its ability to infect and multiply within a host plant. In contrast, the pathogenicity of the nematodes was substantially higher after pre-culture on host plants. The infection rates were negatively correlated with plant vigor and chlorophyll content.

What can be the reason for the low pathogenicity after long-term cultivation on carrot callus? It has been known that the nutritional status of the host edaphic factors such as soil texture, moisture, temperature, and interactions with microorganisms can significantly impact the pathogenicity of root lesion nematodes^2,14,15,17,18^. In our experiments, we kept all factors constant except the pre-culture conditions. Unless not caused by a single virulence gene, pathogenicity can vary greatly in response to environmental factors.

Recent studies on *Caenorhabditis elegans* and nematodes of the genus *Pristionchus* and *Strongyloides* indicate several examples of phenotypic changes due to environmental stimuli. However, these studies primarily concentrated on the concept of phenotypic plasticity, which is the ability of an organism to change its phenotypes in response to external stimuli^31–40^. For example, Susoy, et al.^41^ described a surprising instance of macro-evolutionary scale diversification without genetic diversity. Symbiotic nematodes of the genus *Pristionchus* accumulated polyphenism with up to five distinct adult morphotypes per species after colonizing individual figs, which require transmission by pollinating wasps (*Ceratosolen* spp.). In response to diverse environmental cues, *P. pacificus* can generate either a bacterial feeding or a predatory mouth morph^42^. Igreja and Sommer^43^ discovered that sulfatases and sulfotransferases play a role in *C. elegans* and *P. pacificus* development and their behavior in different environments.

Aging processes could affect pathogenicity. Several studies have shown that aging in nematodes may directly influence microbiota and indirectly alter other characteristics, including pathogenicity. Cabreiro and Gems^44^ demonstrated that *C. elegans* in monoxenic culture could exhibit dysbiosis, which is defined as a state of microbial imbalance in the gut that leads to host dyshomeostasis and involves physiological changes in the bacteria caused by extrinsic factors (e.g., environmental stressors, growth conditions) or intrinsic factors (e.g., aging, either of the host or the bacterial population in the gut lumen). Several studies showed that during the development of *C. elegans*, bacteria primarily serve as a food source throughout development because the pharyngeal grinder completely crushes them. In young adults, bacteria that have escaped the action of the grinder grow and develop a community inside particular areas of the gut lumen, where they remain symbionts. As the nematodes age, bacteria growing in the intestinal lumen of the nematode become harmful to the host^44–49^. Klass^50^ demonstrated that a change in temperature or food composition might affect the lifespan of *C. elegans* at any stage in the life cycle. Interestingly, the findings revealed that parental age and life duration have a minor influence on the progeny's life span.

Genetic changes might alter the pathogenicity of the nematode. Sexual reproduction increases adaptability and heterogeneity in PPN populations. Accordingly, virulent genotypes may result from sexual multiplication^51^. However, *P. neglectus* is a monosexual species reproducing only by mitotic parthenogenesis^52^. Also, mutations cannot explain the varying pathogenicities in our experiments because the isolates could regain pathogenicity after growing on a host plant, which cannot be explained by genetic changes.

We reason that the tripartite pathogen-microbiota-plant interactions are likely to influence pathogenicity. PPNs are associated with intricate microbial communities^22–24,53–56^. Changes in native microbiota can alter the nematode's lifetime and survival rate. Dirksen, et al.^22^ showed that bacterial communities such as *Pseudomonas*, *Stenotrophomonas*, *Ochrobactrum*, and *Sphingomonas* improve the fitness of *C. elegans* under both normal and stressed situations. Furthermore, they showed that several *Proteobacteria* may enter the *C. elegans* gut and that an *Ochrobactrum* isolate can survive in the intestines under stressful conditions.

Host-microbiota-pathogen interactions in *C. elegans* have been studied in artificially generated tripartite interactions and by studying native microbiota. Stevens, et al.^57^ showed that *Enterococcus faecalis* in *C. elegans* behaves as a defensive mutualist supporting host survival in the presence of pathogenic bacteria such as *Staphylococcus aureus*. Their findings indicated that direct and indirect tripartite interactions would most likely occur. Non-competitive effects, such as immunopathology, immunosuppression, and microbiota-mediated tolerance, considerably affect competitive interactions^27,57^. Elhady, et al.^23^ investigated the nematode-associated microbiome between infective stages of *Meloidogyne incognita* and *Pratylenchus penetrans*, which attaches to the nematode's cuticle or surface coat. They found that *Betaproteobacteria*, *Bacilli, Actinobacteria,* and the fungal genera *Malassezia*, *Aspergillus,* and *Cladosporium* were most abundant. The composition of microbial communities on the nematode's surface depended on the soil's microbial community. The highly unique attachment of microorganisms to PPN infective stages in soil suggested an ecological purpose for this association and may be associated with soil suppressiveness towards them. Nuaima^26^ reported that populations of *P. neglectus* from different geographical regions differed in their soil bacterial communities adhering to their cuticles, such as *Rothia* sp. These bacteria decreased the nematode's ability to penetrate the host plant. We reason that long-term cultivation on carrot callus alters the microbiome of *P. neglectus*. In contrast, the cultivation on host plants, such as barley, maintains a microbiome needed for the infection.

Nematodes may use microbial metabolites or exploit host defense depletion and changing environments to trigger infection^27,57^. Metabolites produced by the microbiota may serve as an energy supply, allowing for enhanced virulence, fast development, and higher pathogenicity^27,57^. Hayes, et al.^58^ reported that hatching of the mouse parasite *Trichuris muris* was triggered by bacterial surface structures known as fimbriae^58^. King, et al.^59^ reported that *Enterococcus faecalis* living within *Caenorhabditis elegans* protects the nematode against infection by a more virulent pathogen. A similar study on the native microbiota of *C. elegans* showed that native microbiota might produce necessary nutrients for *C. elegans* and alter the nematode’s fitness^60^. MacNeil^61^ reported how native microbiota alters the development, body size, and fertility of *Pristionchus pacificus* by stimulating the neuronal expression of TGF-β ligands. Another study showed that the native microbiota provides nutrients that dramatically promote the life history of *P. pacificus* by regulating a neuroendocrine peptide in sensory neurons^62^.

The host plant’s physiological status can also influence the nematode’s population structure, sex ratio, and likely pathogenicity^63–65^. In cyst and root-knot nematodes, the sex differentiation is epigenetically determined to some extent, and under conditions of crowding or poor nutrition, the frequency of males increases^64–66^. An adequate diet is essential for the nematode’s development as it supplies the necessary components for cell growth, chemical energy to drive cellular processes, and vital nutrients that the organism cannot produce in sufficient quantities^67,68^. Emerya, et al.^69^ demonstrated that smaller, less productive plants could reduce soil nematodes’ abundance and density by decreasing the belowground biomass. Differences in the plant’s nutritional status can also change belowground resources and influence the composition of soil nematode communities^64,70^. Cortois, et al.^70^ used the shoot carbon-to-nitrogen (C/N) ratio to measure resource quality. They reported that the PPN abundance is influenced more by resource quality than quantity. In another study, the soybean cyst nematode, *Heterodera glycines*, while capable of penetrating specific plant species roots, failed to reproduce successfully if faced with unfavorable conditions, possibly due to the plant’s nutritional status^67,71^. Moreover, the metabolite composition of the host plant has also been analyzed. Viketoft, et al.^72^ observed significant variations in nematode community diversity and composition among species within the same functional groups, such as grasses, legumes, or forbs, potentially due to variances in plant metabolites.

Our data demonstrate that the pathogenicity of *P. neglectus* is restored after infection on a living host such as barley. Therefore, we propose interrupting *P. neglectus* cultivation on carrot callus by host plant infections to prevent pathogenicity decline (Fig. 1). This is mainly relevant for plant breeding, where standardized inoculum is needed for selecting resistant genotypes among large segregating populations.

Moreover, the pathogenicity of root lesion nematodes depending on growth under different environments deserves further investigation. In vitro and in vivo experiments with different *P. neglectus* isolates and microbial communities can reveal details about the interaction between microbiome and nematode^57,73^.

## Materials and methods

### Plant material and greenhouse infection tests

The barley cultivar Valentina and the wheat cultivar Machete were used in this study. Both are susceptible to *P. neglectus*^74–76^. Three hundred plants of each variety were inoculated with different isolates of *P. neglectus* with 30 repetitions in each treatment and repeated twice in a completely randomized design, essentially as described by Keil, et al.^76^ and Fatemi, et al.^30^. Plants were grown in the greenhouse in plastic cylindrical tubes 4 cm in diameter and 15 cm in height, filled with heat-sterilized sand (Probau® Quarzsand eco, grain size: 0.1–0.4 mm) under long-day conditions (16 h light) at 23 °C during the day and 18 °C at night with supplemental light (Son-T Agro 400W, Koninklijke Philips Electronics N.V., Eindhoven, The Netherlands). Plants were irrigated twice a week from the bottom of the tubes with a nutrient solution (Supplementary Table 1), as described by Marshall and Ellis^77^. The nutrient solution was supplied from a 100-L tank and renewed monthly to avoid changes in nutrient concentrations.

Plants were inoculated with different *P. neglectus* isolates that had been cultured under different conditions. In each treatment, 1000 nematodes from specific isolates were used as an initial inoculum after visual counting under a stereo microscope.

### Nematode culture conditions

The *Pratylenchus neglectus* population PnGLS4 originating from farmer’s fields near Groß Lüsewitz, Germany, was kindly provided by the Institute for Epidemiology and Pathogen Diagnostics, Julius Kühn-Institute, Braunschweig, Germany, as described in^30^. *P. neglectus* isolates were produced on carrot calli and living host plants (Table 2). The nematodes were multiplied on carrot calli as a monoxenic culture^7,9^. Carrot discs were surface sterilized by heating and kept in the dark at 25 ± 1 °C for one week. Then, nematodes were sterilized with streptomycin sulfate (0.1%), and 200 nematodes at different stages of development were placed on each disc. Each Petri dish containing one carrot disc was sealed with parafilm and stored in the dark at 25 °C. The carrot disc cultures were checked every two weeks for nematode reproduction and any contamination with fungi and bacteria under a stereo microscope. On average, the multiplied nematodes were transferred to a new carrot disc every three months. Finally, the nematodes were extracted from carrot discs using a misting chamber^30,76^.

Nematodes were cultivated on carrot callus (cultivation on Carrot Callus, CC) for more than 96 weeks (CC1), 96 weeks (CC2), and 48 weeks (CC3). LHP contained nematodes extracted directly from Living Host Plants, and non-inoculated plants were used as controls. Alternatively, after eight weeks of cultivation on plant roots, nematodes were obtained from barley and wheat roots (cultivation on Plant Roots, PR) infected with CC1, CC2, CC3, or LHP isolates. Before inoculation, nematodes were counted, and their mobility was inspected under a binocular microscope (Fig. 1 and Table 2). Before inoculation, each isolate was examined under the microscope to measure morphological characters and the demanian indexes^2,78^.

### Phenotypic measurements

Leaf chlorophyll contents were determined by a Dualex instrument (Force A, Paris, France) described by Casa, et al.^79^. Dry shoot and root weight were measured using a precision scale after harvesting the plant samples and allowing them to dry completely to determine their respective biomass.

### DNA isolation, PCR, and real-time quantitative PCR

After harvesting, root samples were divided into two groups; one half was placed in a freeze dryer for total DNA isolation and RT-qPCR, and the other half was placed in a misting chamber to extract the nematodes and for visual counting using a stereo microscope. Total DNA was isolated from freeze-dried infected roots. RT-qPCR was performed with the Rad CFX Connect^TM^Optics Module Real-time PCR detection system using the species-specific Neg1 primer combination described by Fatemi, et al.^30^.

### Statistical analyses

The RT-qPCR data were analyzed using the Bio-Rad CFX Manager software version 3.1. The amplification efficiency (E) was calculated from the slope of the quantification cycle (Cq) values (y-axis) and log picograms (log pg) of DNA (x-axis) using the equation E = (10^(1/–m)^ – 1) × 100, where *m* is the slope^80^. The reproduction rates, Pf/Pi values, were calculated by dividing the final number of nematodes (Pf) by the number of nematodes in the initial inoculum. The ANOVA was performed with the "Agricolae" program package in R Studio software, version 4.1.0., and significant differences between groups were calculated by a Tukey test (p < 0.05).

### Supplementary Information

Supplementary Information.

## Data Availability

The authors declare that data of this study are available from this manuscript and its supplementary information files. Upon request, more data, information, and materials used in this study are available from the corresponding authors. All methods were carried out following relevant guidelines. The materials used for this study were purchased through standard commercial paths, and no special permits were required.
