# Supplementary material for: Pathogenicity of the root lesion nematode Pratylenchus neglectus depends on pre-culture conditions
Source: Sci Rep. 2023 Nov 10;13:19642. doi: 10.1038/s41598-023-46551-9 (PMC10638436; doi:10.1038/s41598-023-46551-9)
Supplement: Supplementary file 1 — Supplementary Information. [file 41598_2023_46551_MOESM1_ESM.pdf]

Pathogenicity of the root lesion nematode *Pratylenchus neglectus* depends on pre-culture conditions

Ehsan Fatemi[1] and Christian Jung[1*]

[1]Plant Breeding Institute, Christian-Albrechts University Kiel, Germany

[*]Corresponding author email: c.jung@plantbreeding.uni-kiel.de

Supplementary data

Supplementary tables

Nr.	Name	Composition	1 liter	10 Liter	100 Liter	Company
1	Calcium nitrate tetrahydrate ≥98% pure	$Ca(NO_3)_2.4H_2O$	1.41	14.05	140.15	Roth
2	Ammonium nitrate ≥98% p.a ACS	NH_4NO_3	0.17	1.65	16.50	Roth
3	Dipotassium phosphate ≥98% Anhydrous	K_2HPO_4	0.22	2.23	22.25	Roth
4	Potassium sulfate, ≥98 % Cryst.	K_2SO_4	1.50	15.00	150.00	Roth
5	Magnesium sulphate heptahydrate, ≥99 %, p.a., ACS	$MgSO_4.7H_2O$	0.96	9.60	96.00	Roth
6	Ethylenediamine tetraacetic acid iron(III) sodium salt-Trihydrate, ≥98 %, for synthesis	FeNaEDTA	0.0745	0.7450	7.4496	Roth
7	Manganese(II) sulphate monohydrate, ≥99 %, p.a., ACS	$MnSO_4. H_2O$	0.0129	0.1286	1.2864	Merck
8	Boric acid ≥99.8 %, p.a. ACS ISO	H_3BO_3	0.0057	0.0566	0.5664	Roth
9	Copper (II) - sulfate-pentahydrate, for analysis. ACS ISO	$CuSO_4.5H_2O$	0.0012	0.0125	0.1248	Merck
10	Zinc sulfate heptahydrate, for analysis, analysis	$ZnSO_4.7H_2O$	0.0012	0.0125	0.1248	Merck
11	Sodium molybdate- Dihydrate	$Na_2MoO_4.2H_2O$	0.0005	0.0048	0.0480	Merck
	Tap water (Liter)		1	10	100	
	Adjust the pH value with sulfuric acid 98% (Approx. 8ml)	pH	6	6	6	

Supplementary Table 1: The nutrient supplies were used to support cereal plant development as described by Marshall and Ellis 1998.

Supplementary figures

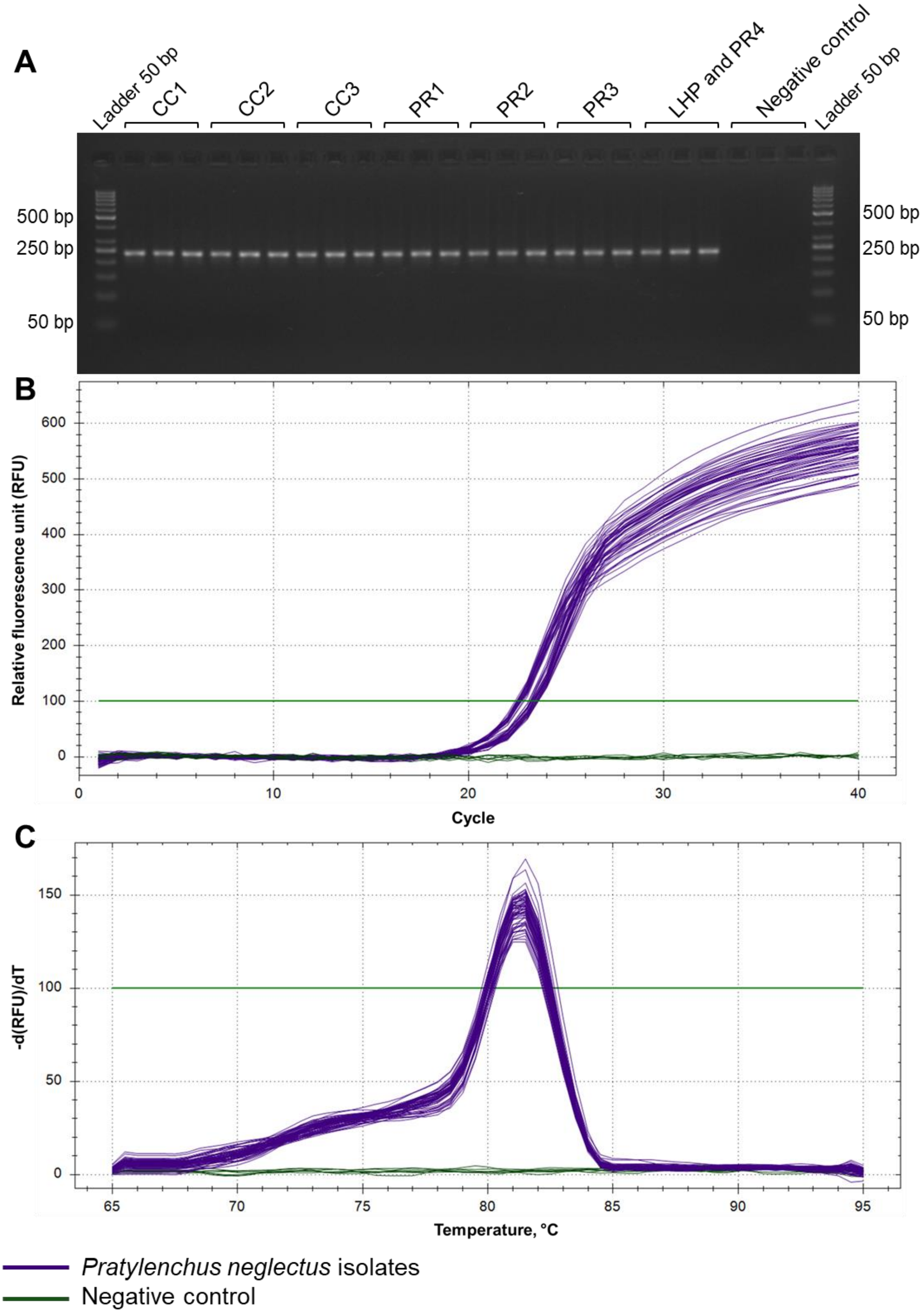

Supplementary Figure 1: PCR and RT-qPCR results with total DNA of cereal plants inoculated with different *P. neglectus* isolates. The primer combination Neg1 was used (Fatemi et al. 2023). (A) Agarose gel electrophoresis (3%, 80V for 60 minutes) with PCR fragments amplified with DNA from different isolates. (B) RT-qPCR amplification curves for seven *P. neglectus* isolates. (C) The melting curve peak of the *P. neglectus*-specific amplicons is at 81.5°C. All samples were analyzed using three technical replicates.

Supplementary figures

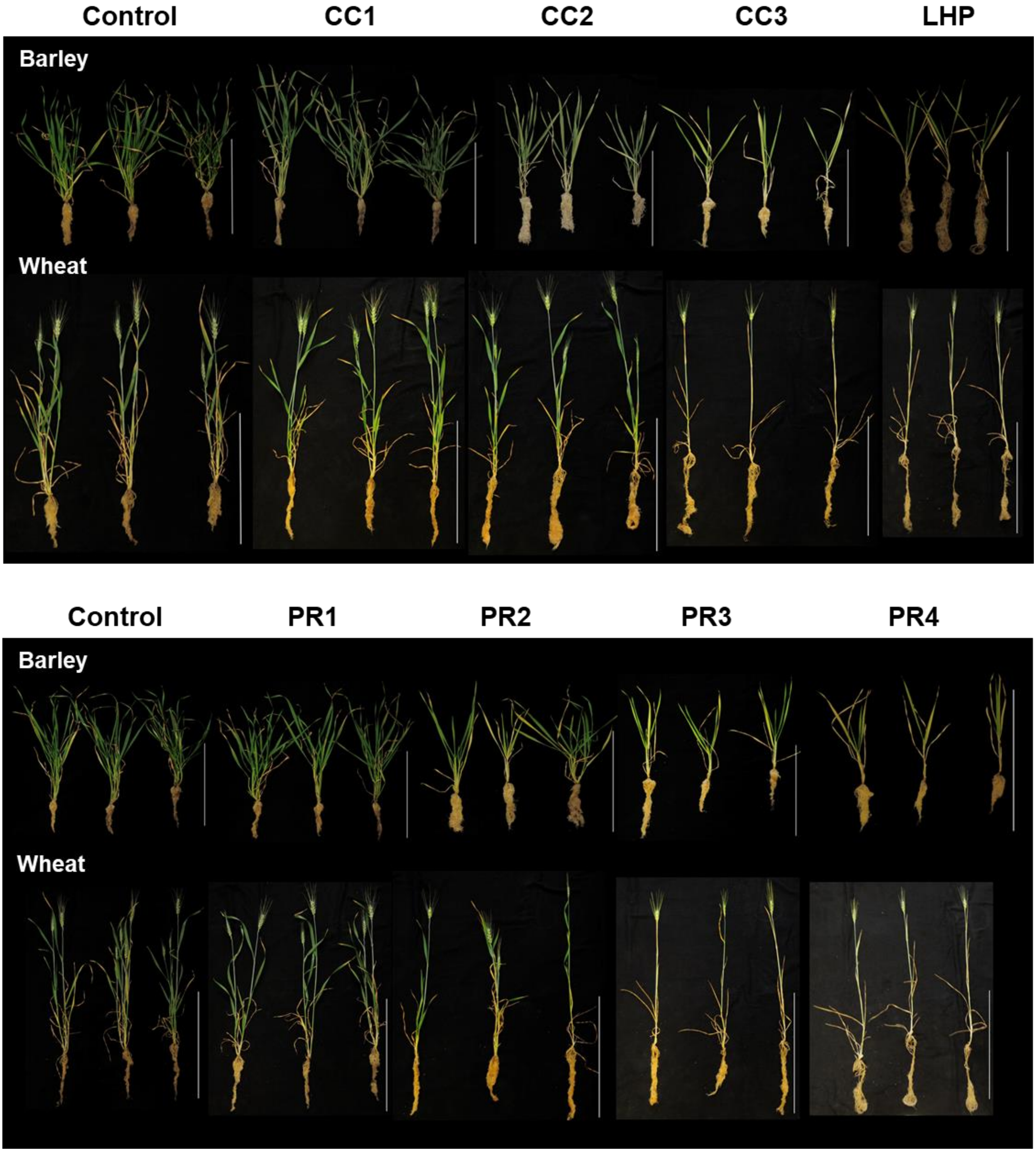

Supplementary Figure 2: Barley and wheat plants from the greenhouse experiment eight weeks after inoculation. Roots were harvested ten weeks after sowing. Plants were infected with 1000 nematodes from different *P. neglectus* isolates. Scale bar: 30 cm.
